# Evaluation of an Artificial Mouth for Dental Caries Development

**DOI:** 10.3390/microorganisms11030628

**Published:** 2023-02-28

**Authors:** Bennett T. Amaechi, Parveez Ahamed Abdul Azees, Rayane Farah, Fatemeh Movaghari Pour, Andrew M. Dillow, Chun-Yen Lin

**Affiliations:** Department of Comprehensive Dentistry, University of Texas Health San Antonio, San Antonio, TX 78229, USA

**Keywords:** microbial caries model, artificial mouth, demineralization, remineralization, biofilm

## Abstract

This study validated a microbial caries model (artificial mouth) for dental caries development to determine the optimal time to create early caries suitable for evaluation of the efficacy of caries therapeutic agents. In all, 40 human enamel blocks were placed in an artificial mouth at 37 °C and 5% CO_2_ and were exposed to brain heart infusion broth inoculated with *S. mutans* in continuous circulation (0.3 mL/min). The culture medium was replaced three times daily. Samples were exposed to 10% sucrose for 3 min, 3 times daily to promote biofilm growth. Five samples were harvested from the chamber after 3, 4, 5, 6, 7, 14, 21, and 28 days. At the end of experiment, samples were assessed visually by ICDAS criteria, while lesion depth (LD) and mineral loss (ML) were measured using polarizing light microscopy and transverse microradiography. Data were analyzed by Pearson correlation, ANOVA, and Tukey comparison test (*p* < 0.05). Results showed significant and strong positive correlation (*p* < 0.01) between all variables and biofilm growth time. LD and ML profiles of 7-day lesions seem to be the most suitable for remineralization studies. In conclusion, using the evaluated artificial mouth, early-stage caries suitable for products’ evaluation studies was produced within 7 days of exposure to microbial biofilm.

## 1. Introduction

Dental caries is a multifactorial disease, and its development is directly related to the presence of cariogenic bacteria on the tooth surface and the frequent consumption of dietary carbohydrates [1]. Dental caries is a widespread cause of pain, which compromises quality of life, also leading to tooth loss, when causative pathogens are not under control [2]. It is now well-established that when detected at an early stage, caries lesions can be reversed by eliminating the cariogenic factors and implementing a remineralization process [3]. For this reason, the development of systems and tools that simulate as closely as possible the clinical situation for the formation of early caries lesions is necessary for the evaluation of caries-detecting devices, anticaries agents, and preventive therapies.

Different in vitro caries models have been developed within the last few decades to evaluate the dental caries process due to the difficulties and high cost of performing in vivo studies [4]. Some physicochemical models such as the pH-cycling method [5,6] have been used; however, this method does not involve the presence of bacteria, which is primordial in the caries process, and as such is unable to test antimicrobial products. As a consequence, microbiological models have been used to simulate the presence of biofilm as one of the main factors in caries development [4,7]. Streptococcus mutans (*S. mutans*) biofilm has been used in these models, and it permits the assessment of biofilm metabolism, the impact of antimicrobial compounds on biofilm, and the anticaries effects of caries therapeutic agents [8,9,10]. Therefore, *S. mutans* bacteria have been used in different microbiological models to produce caries because they are considered the most important cariogenic microorganisms, showing acidogenic and aciduric properties [11] and producing extracellular polysaccharides [12,13] in the presence of sugars. Furthermore, several studies have identified almost 300 growth-supporting genes in the dental caries pathogen Streptococcus mutans, thus suggesting a link of genetic factors to caries susceptibility [14,15]. Reported genetic factors that contribute to caries may include variants in loci for enamel formation, immune response, saliva, taste, and dietary habits [16].

Different *S. mutans* models have been described in the literature using different systems allowing the analysis of the cariogenicity of carbohydrates [17] or antimicrobial effects [8]. However, these models use a constant carbohydrate supply to the bacteria and hydroxyapatite disks instead of tooth substrate. In the oral environment, dental plaque is exposed to substantial amounts of fermentable carbohydrates within a short period of time, and acidogenic microorganisms can use these sugar to synthesize exopolysaccharides, store energy, and produce acid [18]. Following this quick exposure to sugar, the biofilm enters prolonged sugar starvation periods known as “feast-famine” episodes. This event results in microbiological selection, increasing the proportion of aciduric species like *S. mutans* and *S. sobrinus* [1,11].

The artificial mouth system is a multiple-station continuous flow culture model that simulates the biological and physiological activities observed within the oral environment, mainly “feast-famine” episodes, which happen in the presence of dietary carbohydrates with the continuous flow of the culture medium simulating saliva flow [19]. This model has been used in different dental caries studies; however, the caries process and the time required for the production of early caries lesions has not been evaluated. For this reason, the present study assessed the severity, in terms of lesion depth and amount of mineral loss, of dental caries lesions produced over time using the microbial caries model functioning in an artificial mouth.

## 2. Materials and Methods

### 2.1. Dental Sample Preparation and Experimental Grouping

Following the approval of the Institutional Review Board (Approval #: HSC20080233N) of the University of Texas Health San Antonio (UTHSA), unidentifiable sound human dental molars extracted for surgical or orthodontic reasons were collected and stored in thymol until the experiment. Prior to starting the experiment, each tooth was examined with a transilluminator to eliminate samples with cracks, hypoplasia, white spot lesions, stains, and other malformations. Following that, 40 enamel blocks (each 5 mm length × 5 mm width × 2 mm thickness) were produced from 80 teeth using a water-cooled diamond wire saw (Walter Ebner, Le Locle, Switzerland). Then, the blocks were subjected to demineralization by biofilm growth in an artificial mouth model, as described below.

### 2.2. Artificial Mouth Model

**Figure 1:**
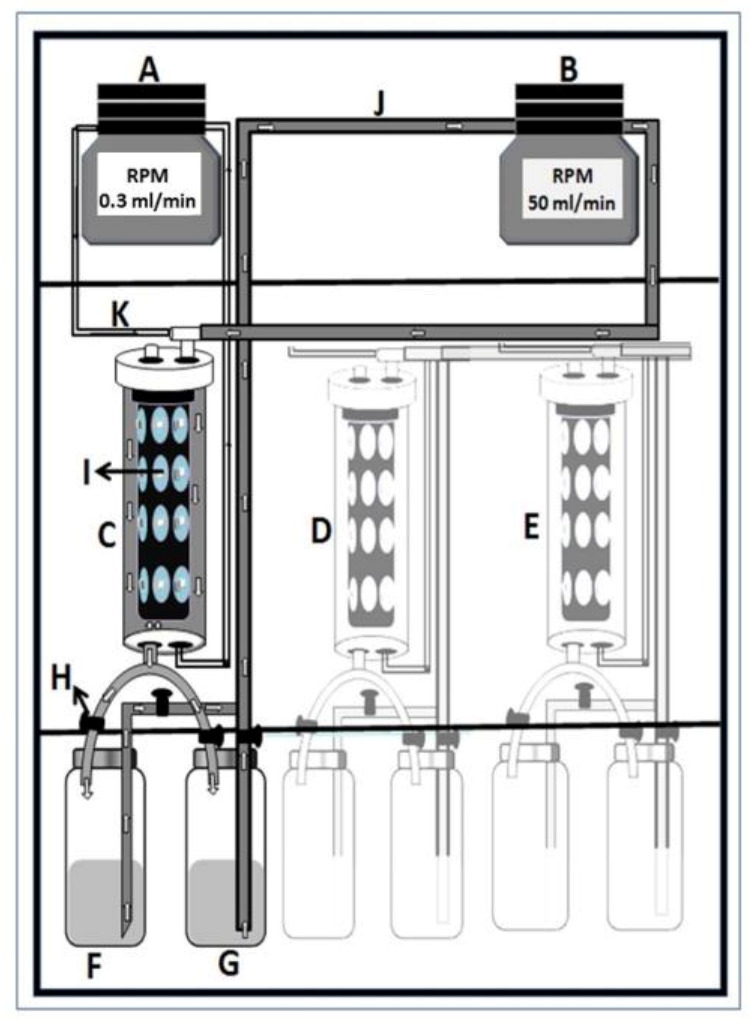
Diagrammatic illustration of the artificial mouth setup housed in a reach-in CO_2_ incubator. (**A**)—programmable broth and sucrose circulation pump; (**B**)—programmable filling and emptying pump; (**C**)–(**E**)—oral chamber housing experimental teeth; (**F**)—broth reservoir; (**G**)—sucrose reservoir; (**H**)—liquid-controlling stopper; (**I**)—cylindrical acrylic rod with tooth blocks; (**J**)—broth and sucrose pumping tube; (**K**)—broth-circulating tube.

The continuous flow biofilm model or microbial caries model (MCM) is composed of multiple stations of continuous flow culture compartments that act as an artificial mouth [19]. It is designed to simulate the natural biological activities occurring within the oral environment (Figure 1). Each station consists of a compartment housing (i) a clear acrylic rod with indentations for mounting either a tooth or tooth blocks; (ii) a head assembly with three lines for supply of simulated oral fluid (SOF), nutrients, and experimental reagents, as well as for inoculation of the compartment with either single- or mixed-organism consortium; and (iii) access for plaque sampling and electrode insertion for pH monitoring. All components of the system are sterilized by autoclaving and aseptically set up. The SOF used in this system is Bacto™ Brain Heart Infusion broth with the pH adjusted to 7.0. The broth is flowed continuously through the compartments at individually controlled flow rates (0.3 mL/min) via digital programmable pumps. A complete circulatory system is established by a to-and-return flow line from the chamber back into the reservoir. The reservoir content is replaced three times daily with fresh media. In addition, 10% sucrose is supplied (flow rate 0.7 mL/min) 3 times daily for 3 min per episode to simulate meals and pH cycling. Following each exposure to sucrose, a sterile phosphate buffer solution (PBS) is run through the circulation to remove residual sugar. All fluids flow uniformly as a thin film over the surface of the rod. The entire assembly is housed inside a reach-in CO_2_ incubator maintained at 5% CO_2_ and at a constant physiological temperature of 37 °C.

### 2.3. Bacteria and Growth Conditions

The glycerol stock culture of *Streptococcus mutans* was revived individually on BD BBL™ Brain Heart Infusion Agar (BHIA) (Fischer Scientific, Greenville, NC, USA) plates and incubated for 24 h at 37 °C. Following incubation, a loopful of individual colonies was transferred from BHI agar plates to tubes containing 10 mL BBL™ Brain Heart Infusion Broth (BHIB) and was incubated for 24 h at 37 °C in a shaking incubator at 150–200 rpm to attain an active exponential growth-phase culture. The stationary growth phase of bacteria was diluted into a final OD600 of approximately 0.06. For further assay, the stationary-phase culture was re-inoculated into BHI broth at an initial turbidity of 0.05 (Spectronic 20; Bausch & Lomb, Rochester, NY, USA), and biofilm formation was performed.

### 2.4. Experimental Procedure and Biofilm Growth

Using heavy-duty putty, the tooth blocks assigned to the experimental groups were embedded in the indentations on the surface of the clear acrylic rods in the culture compartments (10/compartment). The surfaces of the blocks were flushed with the surface of the acrylic rod to permit a streamlined flow of fluids, while only the exposed enamel surface was available for plaque growth and subsequent demineralization. The system was operated as described above by continuous circulation of BHIB separately through each compartment, while 10% sucrose was supplied 3 times daily for 3 min in each episode. The pH of plaque in each compartment was monitored at non-feeding time to check the maintenance of neutrality by CO_2_. The change in plaque pH following sucrose supply was also monitored to confirm pH fall under sucrose challenge and the establishment of Stephan’s curve. Dental plaque growth on tooth blocks on day 1 was initiated by the circulation of pasteurized human whole saliva through the chamber for 30 min to initiate acquired salivary pellicle formation. Following pellicle formation, BHIB inoculated with Streptococcus mutans (NCTC 10449, ATCC, Manassas, VA, USA) culture (broth to inoculum ratio 10:1) was circulated for 90 min to allow bacteria colonization of the pellicle. After this adhesion period, the BHIB was replaced with fresh non-inoculated BHIB. The experiment was performed for 28 days, and the culture medium was changed for fresh non-inoculated media three times daily. On the third day, 2 tooth blocks (not part of the study samples) were harvested to test for plaque formation. The presence of visible biofilm was established by visual examination following staining with plaque-disclosing fluid. Within the 28 days of the experiment, five blocks were harvested after 3, 4, 5, 6, 7, 14, 21, and 28 days. The collected samples were rinsed with a distilled water jet to remove the biofilm and stored at 4 °C until analysis.

### 2.5. Visual Analysis

The enamel surface of each block was examined by an examiner, who was calibrated on the use of the International Caries Detection and Assessment System (ICDAS) criteria for caries scoring [20]. The examiner used the ICDAS caries assessment criteria to gauge the caries lesion severity on each block. The scoring criteria according to the ICDAS were: score 0: sound tooth surface; score 1: first visual change (opacity or discoloration) in enamel hardly visible on the wet surface but distinctly visible after air drying; score 2: distinct visual change (opacity or discoloration) in enamel, visible without air drying; score 3: localized enamel breakdown without visible dentin; score 4: underlying dark shadow from dentin without cavitation; score 5: distinct cavity with visible dentin; and score 6: extensive distinct cavity with visible dentin. The enamel samples were examined under the same ambient conditions with the aid of a light reflector and a dental air/water syringe. Each tooth surface was firstly examined wet and secondly after 5 s of air-drying, as recommended in the ICDAS procedure.

### 2.6. Transverse Microradiography (TMR) Analysis

After completion of the visual examination, each dental block was carefully sectioned through the center of the enamel surface, using a diamond-wire hard-tissue-cutting machine (Well Diamond Wire, Atlanta, GA, USA) to produce an enamel slice approximately 200 µm thick. Then, using adhesive-back polishing cloth in a MultiPrep™ Precision Polishing machine (Allied High Tech, Compton, CA, USA), both sides of the slices were polished to achieve planoparallel surfaces and to reduce their thicknesses to 80–100 µm. Then, with a Phillips X-ray generator system, the polished slices together with an aluminum step wedge (10 steps of 24.5 μm thickness) were microradiographed using type lA high-resolution glass X-ray plates (Microchrome Technology, San Jose, CA, USA). The exposure time was 10 min (20 kV, 10 mA). Following radiography, the plates were processed for 5 min in Kodak HR developer (Rochester, NY, USA) and 15 min in Kodak Rapid Fixer before a final 30 min wash period. After drying, the microradiographs were visualized with a Leica DMR optical microscope linked via a Sony model XC-75CE CCTV camera (Tokyo, Japan) to a 90 MHz Dell™ Pentium Personal Computer (Round Rock, TX, USA). Then, under standard conditions of light intensity and magnification, and along with data from the image of the step wedge, an enhanced image of each microradiograph was analyzed using a TMR program (TMR2006 version 3.0.0.6, Inspektor Research Inc., Amsterdam, The Netherlands) to quantify the parameters of mineral loss (∆Z) and lesion depth (LD) for each caries lesion [21].

### 2.7. Visual Polarized Light Microscopy (PLM) Analysis

Using a Nikon Optiphot^®^ light microscope (Nikon, Tokyo, Japan) with a rotating stage and polarizer, each dental slice, imbibed with water, was examined at a magnification of 10×. The microscope was connected via a Zeiss digital camera (Jena, Germany) to a 90 MHz Dell™ Pentium Personal Computer, housing the Axiovision 4™ image analysis software. The image from the microscope was captured in the computer, and using the software, the mean lesion depth (μm) of the caries lesion was calculated after depth measurement at three different points on the sample.

A power analysis was conducted to determine the sample size. The sample size calculations were performed using the nQuery Advisor software (Statistical Solutions, Cork, Ireland) and were based on previous studies conducted with the microbial caries model [22,23]. To have an 80% power to detect a significant difference among the days of caries formation with an alpha value of 0.0166 (0.05/3) and an effect size of 32.82 (large), a sample size of 3 blocks for each day was needed. However, we used 5 blocks for each group to make provision for damage during processing. The alpha value was set to 0.0166 to allow for multiple comparisons. We used a total of 40 blocks because we planned to harvest 5 blocks after 3, 4, 5, 6, 7, 14, 21, and 28 days, giving 8 harvesting time points, and as such, 40 blocks (5 × 8) were required.

### 2.8. Statistical Analysis

The data were examined for normal distribution through the Shapiro–Wilk test. Data were statistically analyzed using the computer software GraphPad Prism (GraphPad Software, San Diego, CA, USA), with a 95% confidence interval (*p* < 0.05) of difference. Linear regression and Pearson’s correlation coefficient (*p* < 0.05) were conducted on the data to determine the relationship between caries lesion development and the length of time of biofilm growth. In addition, ANOVA followed by the Tukey–Kramer multiple comparisons post hoc test was performed to observe differences among the days of caries formation.

## 3. Results

**Figure 2:**
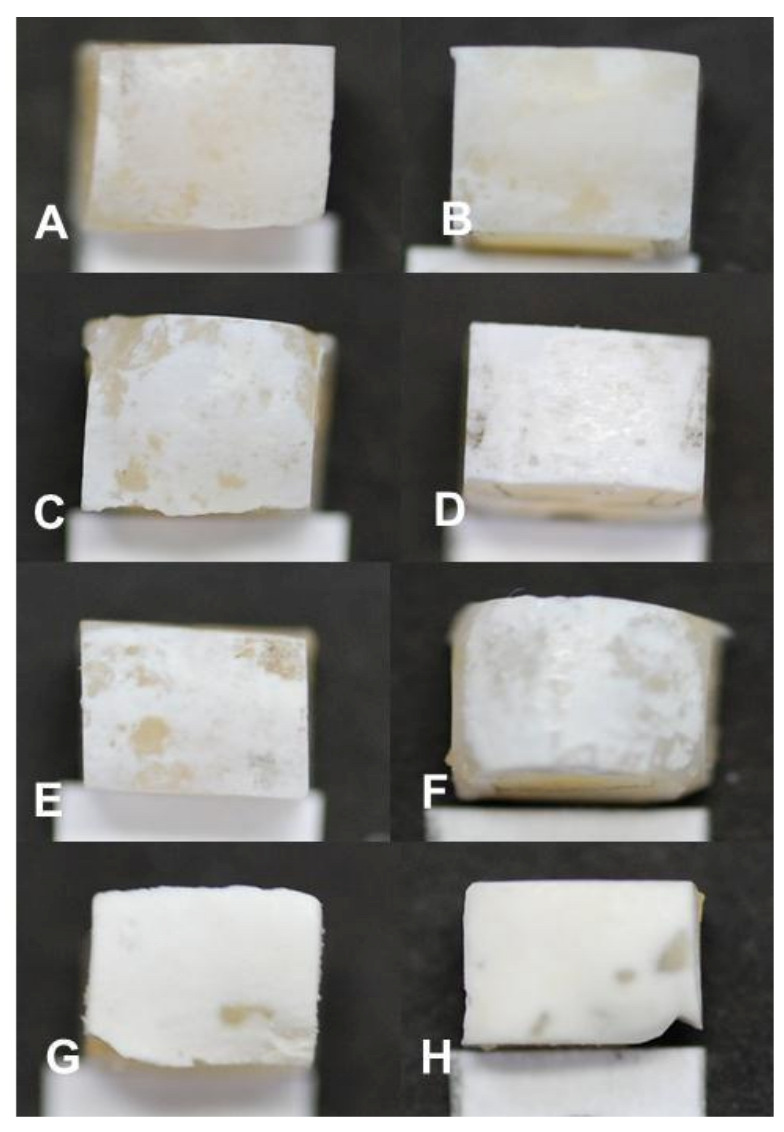
Visual appearance of white spot lesions (early-stage dental caries) produced in the artificial mouth model after 3 (**A**), 4 (**B**), 5 (**C**), 6 (**D**), 7 (**E**), 14 (**F**), 21 (**G**), and 28 (**H**) days of exposure to biofilm growth. Magnification 120×.

With the visual evaluation of the samples, white spot lesions (early-stage dental caries) were observed at every measurement time period in all blocks (Figure 2). Each white spot lesion (WSL) appears opaque and chalky, with a dull (matt) surface when air-dried for 5 s. It feels rough when the tip of a dental explorer is moved gently across the surface. ICDAS score 1 lesions were observed in samples exposed for 3 days (Figure 2A), while 4, 5, 6, 7, and 14 days produced ICDAS score 2 lesions (Figure 2B–F). At 21 and 28 days, the lesions were scored 3 due to observed cavitations with depth limited to the enamel (Figure 2G,H). Visually, the lesion severity increased with the increase in the length of exposure to biofilm (Figure 2).

**Figure 3:**
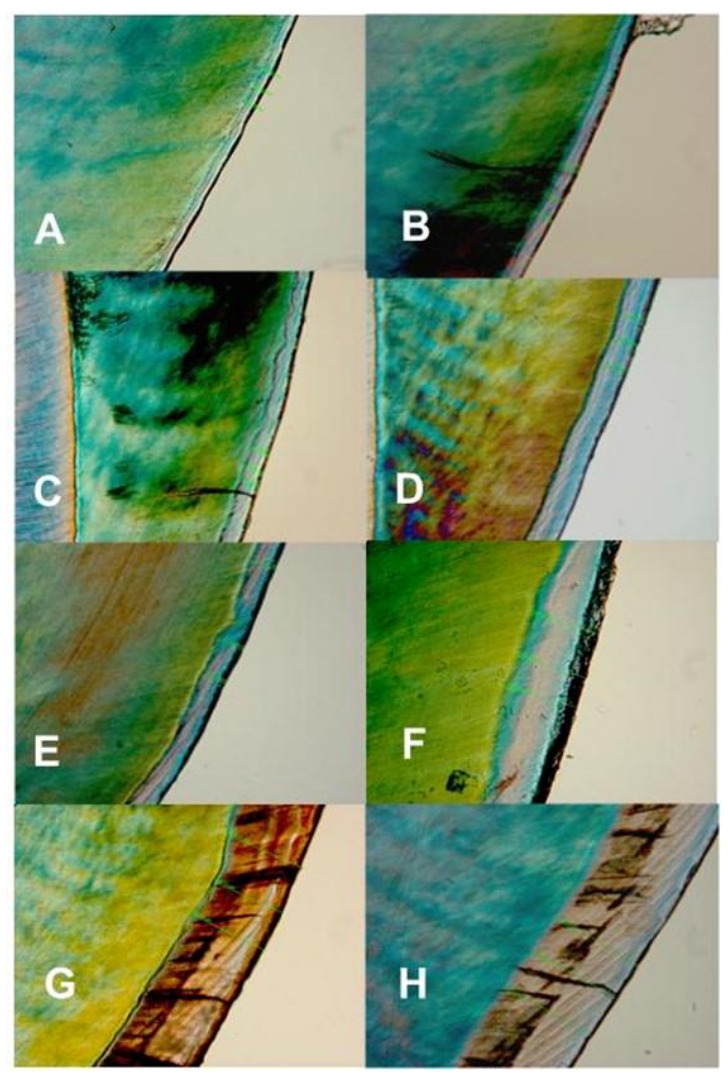
Polarized light microscopy images showing the depth and internal structure of caries lesions produced in the artificial mouth model after 3 (**A**), 4 (**B**), 5 (**C**), 6 (**D**), 7 (**E**), 14 (**F**), 21 (**G**), and 28 (**H**) days of exposure to biofilm growth. Magnification 450×.

**Figure 4:**
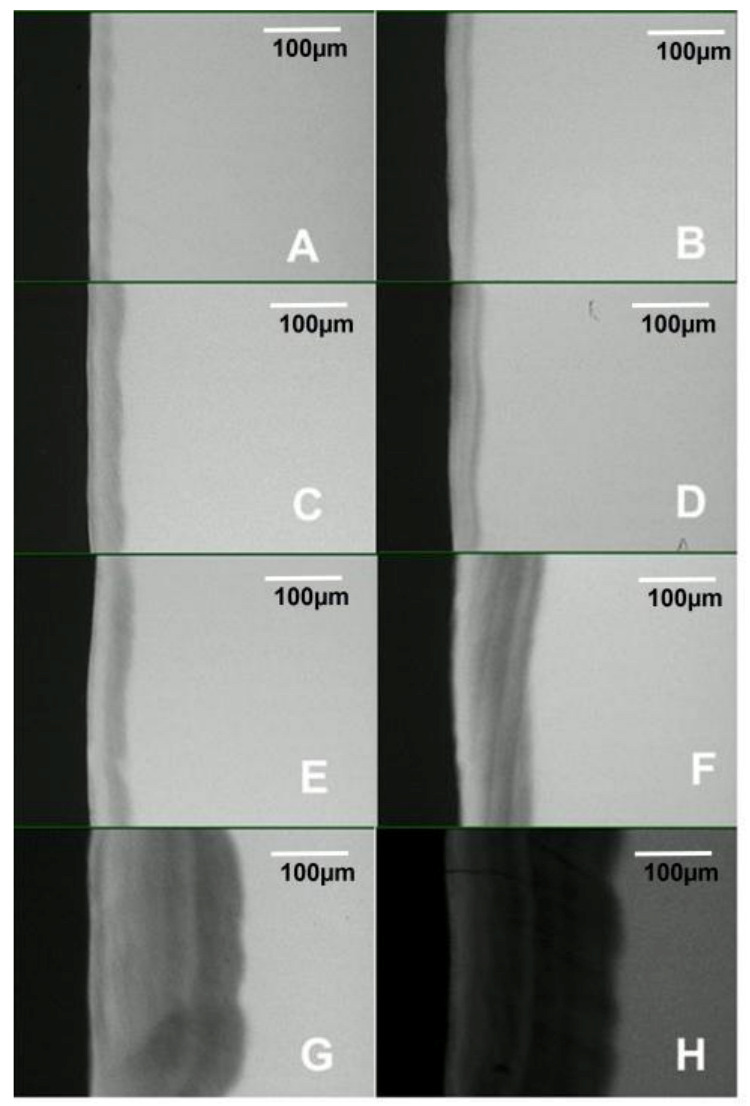
Transversal microradiography images showing the depth and internal structure of caries lesions produced in the artificial mouth model after 3 (**A**), 4 (**B**), 5 (**C**), 6 (**D**), 7 (**E**), 14 (**F**), 21 (**G**), and 28 (**H**) days of exposure to biofilm growth.

**Figure 5:**
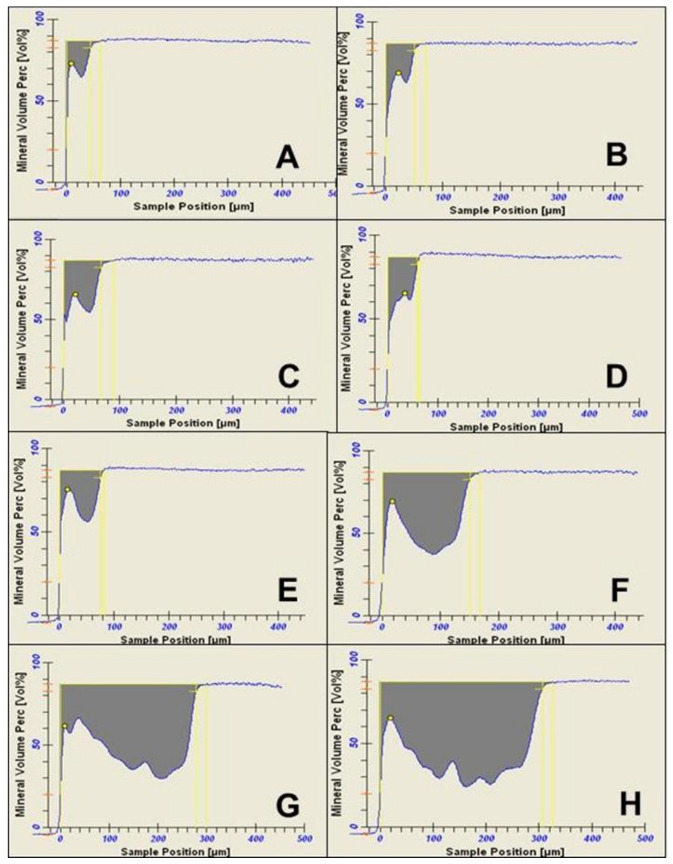
Densitometric and mineral loss profiles, as analyzed by transverse microradiography software, of caries lesions produced in the artificial mouth model after 3 (**A**), 4 (**B**), 5 (**C**), 6 (**D**), 7 (**E**), 14 (**F**), 21 (**G**), and 28 (**H**) days of exposure to biofilm growth.

Pearson’s correlation coefficient indicated a strong positive correlation between the biofilm growth time and lesion depth measured with PLM (R^2^ = 0.9946, *p* < 0.001) and TMR (R^2^ = 0.9929, *p* < 0.001), as shown in Table 1. Also, there was a strong positive correlation (R^2^ = 0.9908, *p* < 0.001) between the biofilm growth time and the amount of mineral loss (Table 1). Both the lesion depth evaluated by PLM (Figure 3) and TMR (Figure 4) and the mineral loss (Figure 5) increased with the increase in the length of exposure to biofilm (Table 1). In all these cases, the analyses showed a similar trend of a linear relationship. A similar trend of linear relationship and a strong positive correlation (R^2^ = 0.9850, *p* < 0.01) were observed between the lesion depth measured with PLM and that measured with TMR (Table 1). Also, at each measurement time point, there was no significant difference between the lesion depth measured with PLM and that measured with TMR (Table 1). Furthermore, there were no significant differences in lesion depth in samples harvested from day 4 through day 7. Similarly, there were no significant differences in mineral loss in samples harvested from day 4 through day 7.

Examination of the PLM and TMR images shows that the WSL has two layers: a relatively sound surface layer and a subsurface demineralization, and this is more defined at 7 days (Figure 2E) but poorly defined at other time points.

## 4. Discussion

It is now understood that early-stage caries is reversible with the practice of better oral hygiene and the use of remineralizing agents [24,25]. With the advent of this knowledge, the last two decades have witnessed a paradigm shift in caries management with more emphasis on minimal-intervention dentistry (MID) [25]. An integral part of MID is the remineralization of caries lesions at their early stage of development, rather than restoration [24,25]. This necessitated the development and evaluation of therapeutic agents to be employed in this remineralization therapy. However, clinical trials of these agents proved to be very costly and time-consuming. For this reason, it is of paramount importance to develop in vitro models for the evaluation and validation of treatment strategies that employ non-surgical modalities, such as fluorides, antimicrobials, sealants, and other anticaries and remineralizing agents [26,27]. Furthermore, the need for a model that will support the identification and clinical staging of the presence, activity, and severity of dental caries is of immense importance in the development and evaluation of caries diagnostic devices to aid clinicians in detecting dental caries at an early stage as well as making a firm diagnosis and treating cases conservatively. In vitro dental caries models have become important tools for these studies, allowing the formation of early caries lesions and the evaluation of the caries-control efficacy of therapeutic agents. These models are expected to simulate the biological and physiological activities that take place in clinical situations as closely as possible. Today, it is well known that dental caries is a biofilm-dependent and sugar-modulated disease. Because biofilm plays a vital role in the etiology of the disease, biofilm models have been developed to study the cariogenicity of dietary sugars, as well as the anticaries effects of substances. For this reason, we developed a microbial caries model (MCM), which is a continuous-flow biofilm model that acts as an artificial mouth [19]. The present study assessed the severity, in terms of lesion depth and amount of mineral loss, of dental caries lesions produced over time using the MCM.

In the present study, using Streptococcus mutans as the microbial agent, we were able to produce dental caries with characteristics consistent with early-stage dental caries: a whitish lesion (WSL) that appears opaque, chalky, with a dull (matt) surface when air-dried, feels rough when the tip of a dental explorer is moved gently across the surface, and histologically has a surface layer and a subsurface demineralization. Early-stage caries lesions (ICDAS scores 1 and 2) are lesions that are not cavitated and as such can be treated by remineralization. These stages of lesions were produced in this MCM within 7 days of biofilm growth on tooth blocks—ICDAS score 1 within 3 days and score 2 within 4–14 days. At 21 days, the lesions started cavitating, with depth limited to the enamel. However, from the day-14 assessment time point, the amount of mineral loss, as shown in Table 1, may not be suitable for the evaluation of the efficacy of a therapeutic agent to remineralize a caries lesion. It has been established that the baseline mineral loss and lesion mineral distribution directly impact caries remineralization by fluoride [28,29]. Although a previous study reported that more demineralized lesions exhibited greater remineralization than shallower ones [26], this report was based on lesions with moderate softening and extreme softening of the tooth tissue and not based on a typical early caries lesion with histological surface and subsurface layers as produced in the present study. With typical early caries lesions with characteristic surface and subsurface layers, it was reported that lesions with low mineral loss were more responsive to fluoride remineralization than lesions with high mineral loss [29]. In the present study, the characteristics of a typical early caries lesion and those required for lesions suitable for remineralization therapy were perfect in 7-day lesions, and for this reason, it is concluded that the MCM evaluated in this study produced caries lesions that better mimic in vivo caries under laboratory conditions and that lesions produced within 7 days of exposure to biofilm growth will be perfect for evaluation of therapeutic agents tailored for a remineralization study.

The observation that TMR and PLM lesion depth results showed a strong linear relationship with each other and with the length (time) of caries formation agrees with the reports of a previous study by Ten Bosch and Angmar-Mansson [30]. These established linear relationships would allow estimating the time needed to obtain specifically sized lesions depending on the requirements of any proposed study. PLM images of the lesions after 21 and 28 days of caries formation showed defects such as fractures and cracks below to the outer enamel surface (Figure 2G,H), which could be caused by the excessive exposure time causing extreme demineralization and weakening of the enamel structure. Also, the profile of mineral loss (Figure 5) of these last two assessment time periods showed an irregular lower edge of the lesion body in comparison with lesions from the other time periods. These two conditions could provoke the overestimation of the mineral loss (in these conditions the software translates the internal enamel defects as highly demineralized areas), suggesting that the limit to produce caries lesions with this model would be 14 days, but 7 days for lesions targeted for remineralization study.

The main goal of the artificial mouth model is to simulate the dynamism found in the oral environment. Regarding the artificial mouth system evaluated in this study, maintaining the continuous circulation of the culture medium (simulating saliva flow) and the use of dental substrate are not common with other biofilm models that employ static culture models [8,31]. The many types of microbial caries models described in the literature can be classified according to different conditions. Such conditions include the number of species used (single versus multispecies), the length of the model (short-term versus long-term), the type of exposure to sugar (continuous versus intermittent), or the type of flow of the culture medium (static or continuous). A static model means the culture medium is not flowing continuously over the growing biofilm [27], so the MCM evaluated in the present study is a continuous model. The advantage of the continuous-flow model over the static model is that due to the culture medium flowing continuously over the substrate and the growing biofilm to simulate the oral fluid (saliva), the acid produced through bacterial metabolism of the sugar does not stagnate within the biofilm and its immediate surrounding medium, keeping the biofilm at a constant pH. The continuous flow of the media ensures pH cycling within the biofilm and the establishment of ‘Stephan’s curve’ with the supply of sucrose, which is associated with dynamism in the caries process. Furthermore, the use of cariogenic challenges by the exposure to sucrose three times per day allows the growth of the *S. mutans* biofilm in “feast-famine” oral conditions. Depending on the objectives of any proposed study, it is possible to change the frequency of sugar exposure or the kind of sugar used. Although the model simulates the demineralization conditions, it is necessary to evaluate whether the mineral content of the culture medium is sufficient to simulate the remineralization process of the saliva after dietary carbohydrate exposure. More studies are necessary to improve the artificial mouth model.

One major limitation of the present study is that the assessments were started on day 3 instead of day 1. This prevented the demonstration of the characteristics of caries lesions produced within 24 to 48 h. Although from the present results it can be deduced that lesions produced within 24–48 h may not be acceptable for product evaluation, they can be used for the validation of caries detection and diagnostic devices. Another limitation of this study is the use of single-organism biofilm, considering that dental biofilm is a mixed-organism consortium with more than one organism involved in caries development. The absence of oral hygiene procedures such as toothbrushing with toothpaste is another limitation of the study, thus making the model represent a high-caries-risk situation of poor oral hygiene condition. Nevertheless, toothbrushing can be incorporated anytime it is needed during the use of the model. However, the strength of this study lies in the design of the artificial mouth model. The model was able to incorporate most of the factors playing parts in the development of dental caries within the oral environment, and as such it can be used as a substitute for in vivo conditions for the evaluation of oral care products as well as caries diagnostic devices.

In conclusion, the present study demonstrated that the evaluated artificial mouth model can be used to produce dental caries lesions that better mimic in vivo natural caries lesions in terms of characteristics. Also, the artificial mouth model can be used to control the profiles (lesion depth, mineral loss, subsurface demineralization) of the caries lesions to be produced. A typical early caries lesion was seen within 14 days, but just 7 days were required for lesions targeted for remineralization study.

## 5. Practical Application

Clinical trials and in vivo studies on human subjects are very costly and time-consuming. The artificial mouth evaluated in this study can serve as a substitute for the human oral environment for the evaluation of oral care products, including caries-preventive therapies. It can also be used for the validation of dental caries-detecting and diagnostic devices. Furthermore, it can help to produce caries lesions of defined characteristics (lesion depth and mineral loss) tailored for a specific study, such as a caries remineralization study, as previous studies have shown that the characteristics of an early caries lesion, particularly the amount of mineral loss and the lesion depth, influence the degree and rate of its remineralization.

## Figures and Tables

**Table 1:** Caries lesion depth (mean ± SD) values assessed with polarized light microscopy (PLM) and transversal microradiography (TMR), and mineral loss with TMR in relation to days of biofilm growth. In rows and columns under the lesion depth, same letters indicate non-significant differences, while different letters indicate significant differences. In the column under mineral loss, same and different letters indicate non-significant and significant differences, respectively.

DAYS	Lesion Depth (µm)				Mineral Loss (vol%·µm)	
	PLM		TMR		TMR	
3	42.24 ± 5.43	a	47.34 ± 6.96	a	798.00 ± 181.58	a
4	65.35 ± 11.44	b	63.56 ± 9.05	b	1642.00 ± 371.04	b
5	71.34 ± 13.22	b	74.88 ± 7.43	b	1928.00 ± 426.23	b
6	88.15 ± 14.99	b	84.40 ± 11.00	b	1952.00 ± 269.67	b
7	93.68 ± 8.15	b	98.74 ± 24.12	b	1977.00 ± 290.22	b
14	189.17 ± 32.72	c	198.20 ± 31.60	c	6542.00 ± 1065.89	d
21	274.87 ± 16.74	d	288.96 ± 30.47	d	11,424.00 ± 1482.85	e
28	336.58 ± 22.80	e	344.28 ± 30.50	e	17,070.00 ± 2065.56	f

## Data Availability

The datasets generated and analyzed during the current study are available upon request from Bennett T. Amaechi (amaechi@uthscsa.edu).
